# Biometry of Anterior Segment of Human Eye on Both Horizontal and Vertical Meridians during Accommodation Imaged with Extended Scan Depth Optical Coherence Tomography

**DOI:** 10.1371/journal.pone.0104775

**Published:** 2014-08-12

**Authors:** Lin Leng, Yimin Yuan, Qi Chen, Meixiao Shen, Qingkai Ma, Beibei Lin, Dexi Zhu, Jia Qu, Fan Lu

**Affiliations:** School of Ophthalmology and Optometry, Wenzhou Medical University, Wenzhou, Zhejiang, China; Medical University Graz, Austria

## Abstract

**Purpose:**

To determine the biometry of anterior segment dimensions of the human eye on both horizontal and vertical meridians with extended scan depth optical coherence tomography (OCT) during accommodation.

**Methods:**

Twenty pre-presbyopic volunteers, aged between 24 and 30, were recruited. The ocular anterior segment of each subject was imaged using an extended scan depth OCT under non- and 3.0 diopters (D) of accommodative demands on both horizontal and vertical meridians. All the images were analyzed to yield the following parameters: pupil diameter (PD), anterior chamber depth (ACD), anterior and posterior surface curvatures of the crystalline lens (ASC and PSC) and the lens thickness (LT). Two consecutive measurements were performed to assess the repeatability and reproducibility of this OCT. They were evaluated by calculating the within-subject standard deviation (SD), a paired t-test, intra-class correlation coefficients (ICC) and the coefficient of repeatability/reproducibility (CoR).

**Results:**

There were no significant differences between two consecutive measurements on either horizontal or vertical meridians under both two different accommodative statuses (P>0.05). The ICC for all parameters ranged from 0.775 to 0.998, except for the PSC (0.550) on the horizontal meridian under the non-accommodative status. In addition, the CoR for most of the parameters were excellent (0.004% to 4.89%). In all the parameters, only PD and PSC were found different between the horizontal and vertical meridians under both accommodative statuses (P<0.05). PD, ACD, ASC and PSC under accommodative status were significantly smaller than those under the non-accommodative status, except that the PSC at the vertical meridian did not change. In addition, LT was significantly increased when accommodation.

**Conclusion:**

The extended scan depth OCT successfully measured the dimensions of the anterior eye during accommodation with good repeatability and reproducibility on both horizontal and vertical meridians. The asymmetry of lens posterior surface and oval-shaped pupil were found during accommodation.

## Introduction

Accommodation is the ability of the eye to change the refractive power to focus on the objects at different distances, which is controlled by ciliary muscle contraction. [1] Among all the accommodation theories, the Helmholtzian theory is the most widely accepted. [2] In order to allow the retina to focus on objects at near distances with clarity, the ciliary muscle contracts, zonular fibres relax, causing the thickness, curvature and position of the crystalline lens to change. [3], [4] Rohen JW. et al. used the anatomical method to confirm this theory and further explained the process of accommodation. [5].

However, the ability of accommodation decreases gradually as age increases. [6] The inevitable decline in accommodative amplitude and characteristic loss of near visual function with age causes presbyopia. Although the occurrence and development of presbyopia is not entirely clear, the most recent evidence suggests that lenticular processes are of key significance. [7] The previous study focuses on the changes of the ocular diopter, but this is not enough to fully understand the entire accommodative system. [8], [9] During the ocular accommodation, the shape and position of the crystalline lens changes, and this was considered to be the most important factor in the power change of the ocular optical system. In addition, changes in the pupil diameter and the anterior chamber dimensions also play an indispensable role in accommodation. Therefore, it is essential to investigate the dynamic variations of the anterior segment configuration dimensions in understanding the characteristics of the ocular accommodation.

Recently, several techniques have been developed for measuring the anterior segment of the eye in vivo, such as the slitlamp assessment, [10] Scheimpflug imaging, [7], [11] A scan ultrasounds,[12] ultrasound biomicroscopy (UBM), [13] and Purkinje reflexes. [14] However, the slitlamp method is entirely subjective, and measurements can therefore vary between examiners. Rotating Scheimpflug images are not corrected for optical distortion and their resolution is relatively low. A scan ultrasound and UBM are both invasive methods and can affect the morphology of the anterior chamber. The Purkinje reflexes cannot directly reflect the ocular optic configuration dimensions.

Optical coherence tomography (OCT) has been widely recognized as a rapid, non-invasive and precise technology for biometric measurements at present. [15]–[17] In our previous study, an extended scan depth spectral domain OCT (SD-OCT) was verified to be a potential method in quantifying changes in the anterior segment dimensions on the horizontal meridian during accommodation. [18] However, the information of the vertical meridian was not mentioned. The goals of this study are to determine the repeatability and reproducibility of this SD-OCT in measuring the anterior segment on both the horizontal and vertical meridians, and to compare the differences of the anterior segment dimensions between the two meridians during the non- and 3.0 diopters (D)-accommodative statuses.

## Materials and Methods

### Subjects

Twenty prepresbyopic volunteers (9 female and 11 male) were recruited from Wenzhou Medical University, with a mean age ± standard deviation of 25.95±2.31 years. The mean spherical equivalent of the subjects was −0.42±0.29 D. None of the subjects had a history of ocular or systemic disease or surgery. This study was approved by the Office of Research Ethics, Wenzhou Medical University.

Written informed consent was obtained from each subject, and all procedures were performed according to the Declaration of Helsinki requirements for research involving human subjects.

### Instruments

Extended scan depth SD-OCT was used to image the ocular anterior segment, which has been previously detailed. [18], [19] In brief, the center wavelength of the super luminescent diode-based light source (Inphenix, IPSDD0808, Livermore, CA) was 840 nm, with a bandwidth of 50 nm. The axial resolution of the system in the eye was approximately 7.5 µm. An extended depth range of 7.8 mm in air was achieved by a custom spectrometer. The total exposure power was less than 1.00 mW, and within the safe range for the human eye, according to ANSI Z136.1. [20].

### Measurements

**Figure 1:**
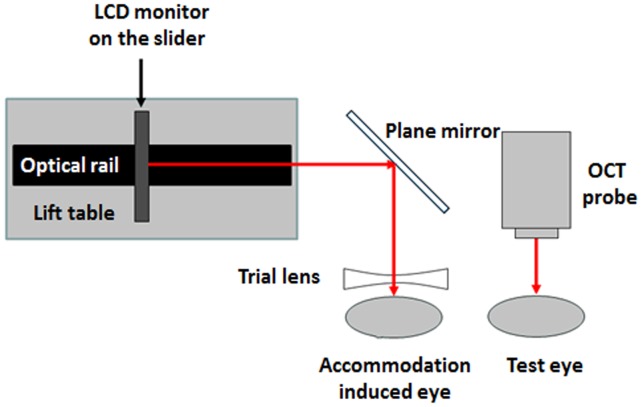
Diagram of the fixation target system. A 1.2 m long optical rail was installed upon a lift table, and placed beside the slit lamp, fixed by a Philips 17-inch LCD monitor on a slider for displaying the target. This LCD monitor was able to move along the optical rail to adjust the distance between the visual target and the human eye, to induce a different stimulus of accommodation. In order to allow the subjects to fix on the target, without being affected by the moving probe, a plane mirror was installed on the slit lamp column, so that the target was projected into the eye after specular reflection.

**Figure 2:**
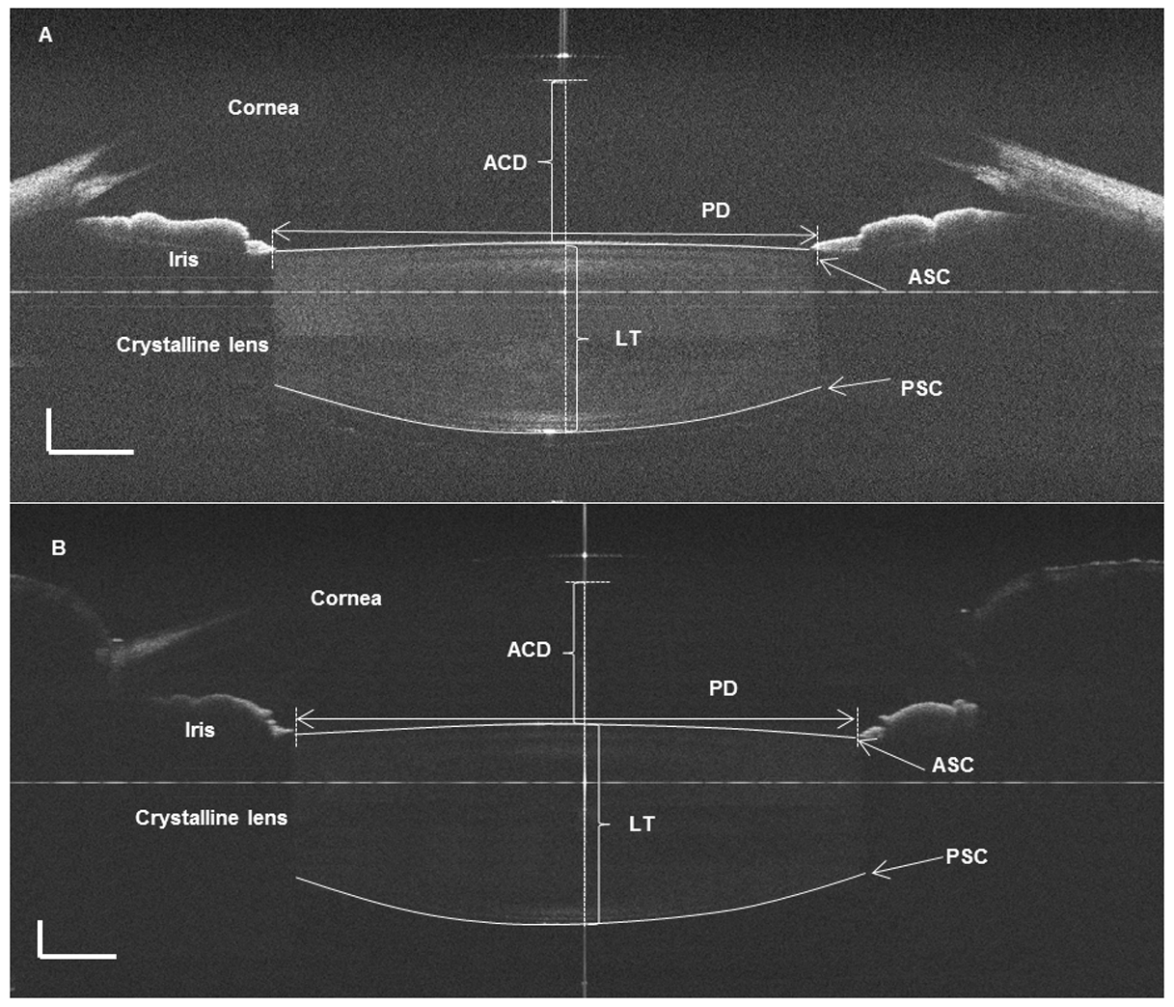
OCT images of ocular anterior segment on horizontal (A) and vertical (B) meridians. The values of dimensional parameters were obtained from the images using custom software. Anterior chamber depth (ACD) and lens thickness (LT) were measured along the perpendicular line. Pupil diameter (PD) was measured along the horizontal line from both ends of the iris. The radii of anterior and posterior surface curvatures of the crystalline lens (ASC/PSC) were measured from the two surfaces of the lens.

**Figure 3:**
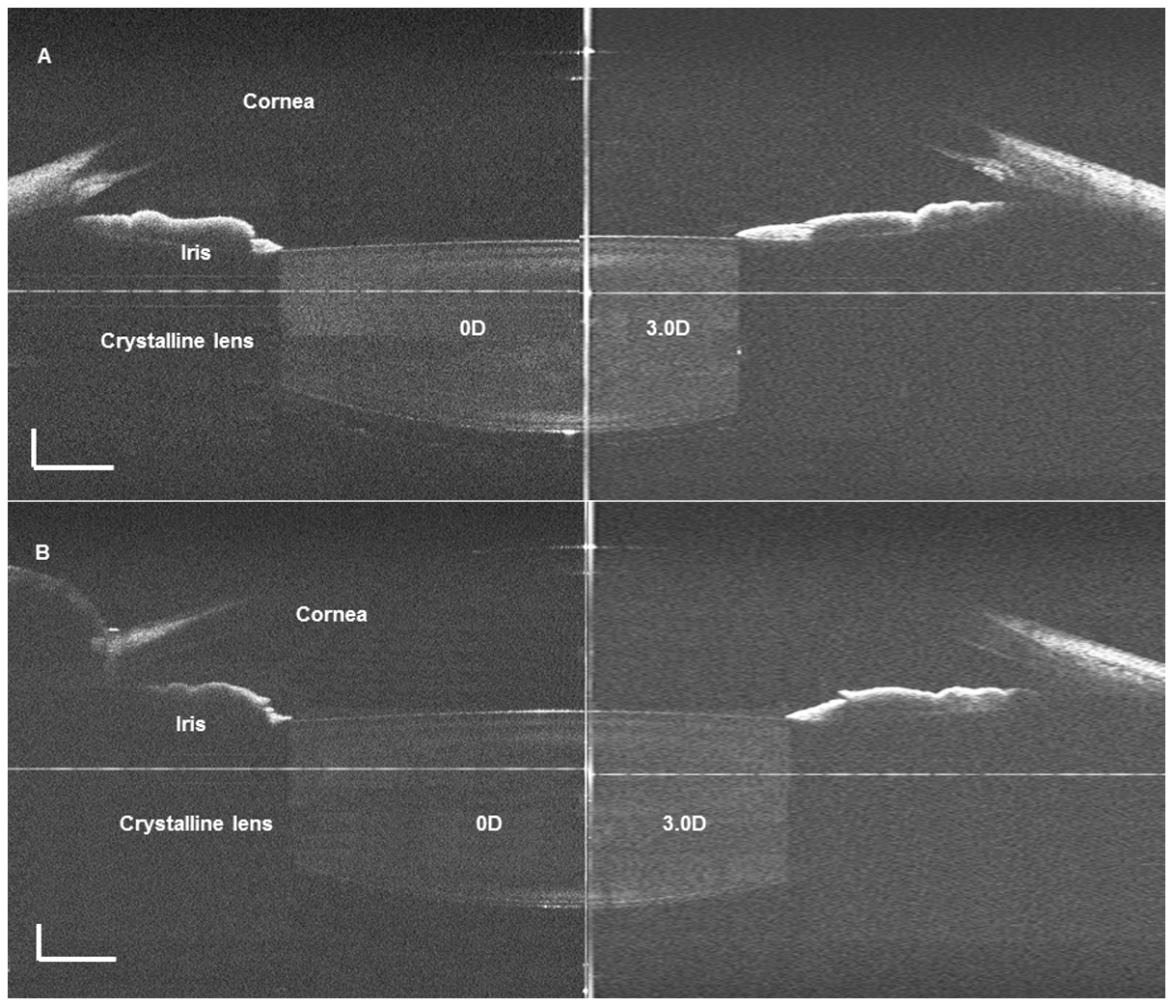
The comparison of OCT images obtained before (left) and after (right) accommodation on horizontal and vertical meridians.

The experimental procedures for each subject were arranged after 10:00AM. Trial lenses were set in front of the left eye of each subject, who were each viewing a target at a non-accommodative and 3.0 D of accommodative demands successively. The right eye was imaged with the SD-OCT by an experienced operator (Fig. 1). For each subject, both horizontal and vertical meridians were taken twice under two different accommodative statuses. A Matlab program was developed for image correction and processing on the RAW OCT images to yield dimensional parameters of the anterior segment of the eye. In total, five anterior segment parameters were obtained with the post-process constructed images in the horizontal and vertical meridians, respectively (Fig. 2). They included pupil diameter (PD), anterior chamber depth (ACD), anterior and posterior surface curvatures of crystalline lens (ASC and PSC), and crystalline lens thickness (LT). As shown in Fig. 3, the anterior segment changed from the non-accommodative status to the 3.0 D-accommodative status.

### Statistical Analysis

Statistical analysis was performed using SPSS for Microsoft Windows (version 18.0, SPSS, Inc.). Results were presented as the mean ± SD. Paired t-tests were used to compare the intra-subject repeatability. All statistical tests were two-tailed. If the P values were less than 0.05, the results were considered statistically significant. Intra-class Correlation Coefficient (ICC) is a measure of correlation for data of repeated measurements. Coefficient of reproducibility (CoR) takes into account the impact of random and/or systematic errors on reproducibility, where the result of reproducibility is more reliable when the CoR is smaller.

## Results

Comparisons of the anterior segment dimensions between the horizontal and vertical meridians during the non-accommodative and 3.0 D-accommodative statuses (Table 1) were revealed. There were significant differences of PD and PSC between the horizontal and vertical meridians under both statuses (P<0.05).

**Table 1:** Anterior Segment Dimensions at the Horizontal and Vertical Meridians during accommodation.

Variables(mm)	Non-accommodative Status		3.0D-Accommodative Status	
	Horizontal Meridian	Vertical Meridian	Horizontal Meridian	Vertical Meridian
PD^a^	5.39±0.75	5.66±0.78*	4.84±0.94	5.37±0.68*
PD^b^	5.38±0.83	5.71±0.67*	4.85±0.94	5.39±0.66*
Diff	−0.01±0.01	0.06±0.07	0.00±0.01	0.02±0.04
ACD^a^	3.15±0.26	3.18±0.27	3.05±0.25	3.06±0.27
ACD^b^	3.16±0.26	3.18±0.27	3.06±0.26	3.07±0.29
Diff	0.01±0.01	0.01±0.10	0.01±0.02	0.01±0.04
ASC^a^	12.21±1.38	11.99±1.64	10.27±1.16	10.48±0.93
ASC^b^	12.05±1.44	11.81±1.09	10.53±1.42	10.45±0.95
Diff	−0.17±0.06	−0.15±0.69	0.27±0.99	−0.02±0.01
PSC^a^	5.62±0.72	6.12±0.62*	5.28±0.56	5.87±0.55* #
PSC^b^	5.65±0.46	5.95±0.56*	5.41±0.44	5.94±0.54* #
Diff	0.03±0.18	−0.16±0.41	0.13±0.44	0.07±0.29
LT^a^	3.77±0.18	3.78±0.17	3.89±0.17	3.89±0.17
LT^b^	3.77±0.19	3.80±0.18	3.88±0.18	3.90±0.18
Diff	0.00±0.01	0.02±0.01	−0.01±0.00	0.00±0.00

a Mean ± SD for the first measurement of the eye.

b Mean ± SD for the second measurement of the eye.

* Statistically significance of the parameters between the two meridians (P<0.05).

# No statistically significance of the parameters during accommodation (P>0.05).

There was no statistical significance of any of the parameters between the two repeated measurements at either meridian under different accommodative statuses (P>0.05).

PD, pupil diameter; ACD, anterior chamber depth; ASC, anterior surface curvature of crystalline lens; PSC, posterior surface curvature of crystalline lens; LT, crystalline lens thickness; Diff, difference between two repeated measurements.

Reproducibility and repeatability of five anterior segment parameters were determined under the different conditions (Table 2). Of all the parameters, there is no statistically significant difference between two repeated measurements on either meridian under different accommodative statuses (P>0.05). The ICC for all parameters ranged from 0.775 to 0.998, except for PSC (0.550) on the horizontal meridian under the non-accommodative status.

**Table 2:** Repeated Measurements of the Anterior Segment Dimensions.

		PD	ACD	ASC	PSC	LT
H-0.0D^a^	CoR(%)	0.235	0.387	0.460	3.273	0.224
	ICC	0.979	0.997	0.927	0.550	0.976
V-0.0D^a^	CoR(%)	1.259	3.108	5.793	6.779	0.185
	ICC	0.973	0.987	0.841	0.850	0.987
H-3.0D^b^	CoR(%)	0.299	0.618	9.502	8.163	0.046
	ICC	0.997	0.998	0.858	0.887	0.991
V-3.0D^b^	CoR(%)	0.712	1.279	0.087	4.885	0.004
	ICC	0.998	0.998	0.775	0.881	0.997

a Repeatability of the variables obtained from two repeated measurements at either meridian in non-accommadative status.

b Repeatability of the variables obtained from two repeated measurements at either meridian in 3.0 D-accommadative status.

PD, pupil diameter; ACD, anterior chamber depth; ASC, anterior surface curvature of crystalline lens; PSC, posterior surface curvature of crystalline lens; LT, crystalline lens thickness; H, horizontal meridian; V, vertical meridian; CoR, coefficient of repeatability; ICC, intra-class correlation coefficient.

The CoR for most of the parameters were presented (0.004% to 4.89%). However, the CoR for ASC (9.50%) and PSC (8.16%) on the horizontal meridian under 3.0 D-accommodative status, and ASC (5.79%) and PSC (6.78%) on the vertical meridian under the non-accommodative status were relatively high.

The values of PD, ACD, ASC and PSC on both meridians under the accommodative statues (Table 1) were significantly smaller than those under the non-accommodative status (P<0.05), except that the PSC did not change on the vertical meridian (P>0.05). In addition, the LT was significantly increased with accommodation (P<0.05).

## Discussion

Optical coherence tomography is a new imaging instrument based on the optical interference principle. Izatt JA. et al. [21] took advantage of OCT to image the anterior segment for the first time in 1994. OCT probes biological tissue backscatter and reflection from the incident light. With this optical interferometric imaging principle, OCT has the advantage of high resolution, fast imaging and noninvasive biopsy-like qualities. Owing to its long scan depth, SD-OCT was used to image the full-range of ocular anterior segments in the present study. Not only can it clearly show the structure of the anterior chamber and pathological changes, but it can also be used to make quantitative analysis. [22], [23] Dimensional parameters of the two accommodative statuses from two meridianal measurement showed good reproducibility and repeatability.

In previous studies, several imaging technologies have been used to quantify the dimensions of ocular anterior segment. Tsorbatzogulou, A. et al. [24] indicated that the lens thickness and anterior chamber depth were 3.776±0.188 mm and 3.578±0.278 mm, respectively using a partial coherence interferometry. Yan, PS. et al. [25] reported 4.56±0.86 mm of pupil diameter and 3.79±0.22 mm of anterior chamber depth with a slit-lamp OCT. Using Scheimpflug imaging technology, Rosales, P. et al. [26] measured ACD, LT, ASC and PSC and gave averaged values of 2.86 mm, 4.06 mm, 10.37 mm, and 5.55 mm, respectively. Hermans, EA. et al. [27] reported that the ASC and PSC were 11.45±1.7 mm and 6.11±1.4 mm, respectively using MRI. Ortiz S.et al. [28] took advantage of three-dimensional (3D) OCT imaging to present that the values of ASC (ranged from 10.27 to 14.14 mm) and PSC (ranged from 6.12 to 7.54 mm). The anterior segment parameters at the horizontal or vertical meridians including ACD, PD, LT and the radii of both surface curvatures of the lens were consistent with previous studies with subjects of comparable age and refractive statuses.

However, in most of previous studies, only one meridian of the anterior eye was imaged. The dimensional parameters of anterior eye were always presented by an average value. In addition, few literatures investigated the dimensional changes of anterior eye during accommodation. This may be due to the technical limitation of imaging methods. OCT is a rapid and non-invasive method with micrometer-scale resolution, and is a suitable tool for investigate the morphology of anterior eye with accommodation. [18] In the present study, we compared the parameters between vertical and horizontal meridians, and did find that there were significant differences in PD and PSC between the two meridians. To the best of our knowledge, this may be the first time assessing the differences in ocular dimensional parameters between vertical and horizontal meridians during accommodation. We suppose these differences should be considered in future studies on accommodation mechanism and visual function.

For instance, significant differences were found in PD between vertical and horizontal meridians. This indicated that the pupil is vertical oval shaped during accommodation. [29]–[30] Thus, if we want to study the effect of pupil on the ocular wavefront aberrations or refractive surgery, pupil area, rather than pupil diameter, may be a more acceptable parameter to represent the pupil size.

Significant differences were found in PSC between the two meridians. The values of ASC and PSC have been measured by several methods both in vivo, [26], [31] and in vitro, [32], [33] and similar results were reported. For instance, Chien CH. et al. [34] made use of polar coordinates and found a parameterization with cosines to prove it to be the most suitable for the human lens surface. Kasprzak HT. et al. [35] proposed an analytical function that describes the complete axisymmetric lens profile in two different accommodative statuses. Hermans, EA. et al. [27] calculated the mean surface area based on eight parts of the lens, measured with 3D MRI, and confirmed the non-symmetric properties of the human lens. Ortiz S. et al. [28] verified lens surfaces to be fitted by biconicoids and Zernike polynomials. Our finding indicated the asymmetry of the posterior surface of the lens, however, this was not found to be true of the anterior surface. This may be due to the growing up of the lens during human's life. Rosen AM. et al. [32] proposed that the anterior part of the lens grows more slowly than the posterior surface. It is possible that with gravity, the down-deposition of the lenticular cells may result in the differences between horizontal and vertical curvatures of the lens.

In the present study, the OCT scans were performed across the corneal apex. However, the lens may tilt and become decentered since it is located in the aqueous humor. Thus it is possible that the apex of lens surface was not captured during OCT imaging. This may explain why the CoRs of ASC and PSC were slightly bigger compared to other parameters. In our future studies, three-dimensional OCT scan is proposed to obtain reconstructed 3D image of the lens. Thus the apex of lens surface can be identified and the radius of lens surface can be calculated more accurately.

In conclusion, the extended scan depth OCT successfully measured the dimensions of the anterior eye in both non-accommodative and accommodative statuses. Good repeatability and reproducibility were presented at both horizontal and vertical meridians. The asymmetry of the posterior surface of the lens and oval-shaped pupil were found during accommodation.
